# Improving the health of people with multimorbidity: the need for prospective cohort studies

**DOI:** 10.15256/joc.2011.1.10

**Published:** 2011-12-27

**Authors:** Stewart W. Mercer, Jane Gunn, Sally Wyke

**Affiliations:** ^1^General Practice and Primary Care, Institute of Health and WellBeing, University of Glasgow, Glasgow, Scotland, UK; ^2^Primary Care Research Unit, Department of General Practice, University of Melbourne, Melbourne, Victoria, Australia; ^3^College of Social Science, Institute of Health and WellBeing, University of Glasgow, Scotland, UK

**Keywords:** long-term conditions, MALT study, multimorbidity, primary care

## The many challenges of multimorbidity

The dramatic rise in long-term conditions (LTCs) represents a major challenge for individuals, families, and healthcare systems worldwide [1]. Due to the scale of this rise, the management of patients with LTCs largely falls within the domain of primary rather than secondary care, at least in countries with well-developed primary care systems. For example, in the UK, which has a comprehensive primary care system based around general practice (trained family physicians working in multidisciplinary teams) and funded by the National Health Service (NHS), primary care contacts account for around 90% of the total activity of the NHS, and patients with LTCs account for 80% of general practice consultations [2]. Effective primary care and community-based management of people with LTCs is thus a top priority [2–6].

Multimorbidity (usually defined as the co-existence of two or more LTCs in an individual, though definitions vary) [7] is the norm rather than the exception for people with LTCs in the UK [8, 9] and elsewhere [10–12], and is related to both increasing age [13] and socio-economic deprivation [9]. Multimorbidity cuts across the vertical paradigms in which most health research is envisaged and funded [14]. Patients with multimorbidity are usually excluded from such research and thus the nature and complex needs of such patients are not well understood and the evidence-base on which to treat them is lacking [14–17].

Our knowledge and understanding of variations in the effect of multimorbidity on issues such as illness burden [18], quality of life and well-being [19], treatment burden [20], and unmet needs [9], remain poor. Furthermore, studies on the quality of primary care [21, 22] and other community-based resources [23] and their impact on patients with multimorbidity are also rare.

## Where should we begin?

Despite the high prevalence of multimorbidity and the key role of primary care in its management, the evidence-base for interventions in this group in primary care is extremely limited [24]. More research on interventions in primary care for people with multimorbidity is urgently needed, but what should these interventions be based on? How do we know what is likely to help?

An important precursor to developing effective interventions is knowledge of the effects of multimorbidity over time and the factors which influence these effects. Prospective cohort studies are the most robust method of studying and describing the natural history and development of morbidity and also for the development and implementation of prognostic models [25, 26]. However, a recent systematic review of prospective cohort studies of multimorbidity in primary care identified only a handful of studies [27]. Although the studies identified provide some useful information, they also demonstrate the significant gaps that exist in knowledge.

To plan future healthcare services and treatment guidelines for those with multimorbidity we must better understand the personal experience, treatment, and health service use, and the psychological, physical, and social factors that influence multimorbidity. Fortunately, several new large cohort studies are underway in Canada and Europe, which will yield important information in the years to come [28–30].

## The MALT study

We have also been working to establish such a cohort in Scotland, a devolved nation within the UK that is responsible for its own health budget and for how NHS services are configured. The Scottish Multiple And Long-Term conditions (MALT) cohort study aims to support essential research on issues such as:

- The natural history and impact of multimorbidity (including predictors of poor outcomes)
- The relationships between multimorbidity and the accessibility, use, and costs of primary care, other health and social care services and community-based resources
- The influence of multimorbidity on patient engagement, activation, and self management
- The process and outcome of clinical encounters over time and factors promoting or hindering the development of preventive (anticipatory) patterns of care
- The quality and safety of care in such patients
- The components and combinations of primary care and other supports that work best in multimorbidity.

**Figure 1:**
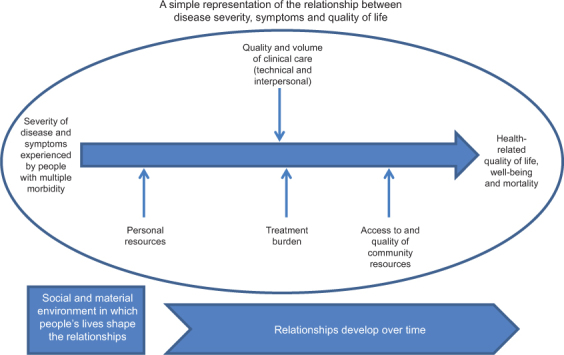
Framework for the Scottish Multiple And Long-Term conditions (MALT) prospective, cohort study.

Additionally, future linked studies could include complex interventions and genetic and environmental predictors of morbidity and poor outcomes in multimorbidity. The framework for the MALT study is shown in Figure 1.

**Figure 2:**
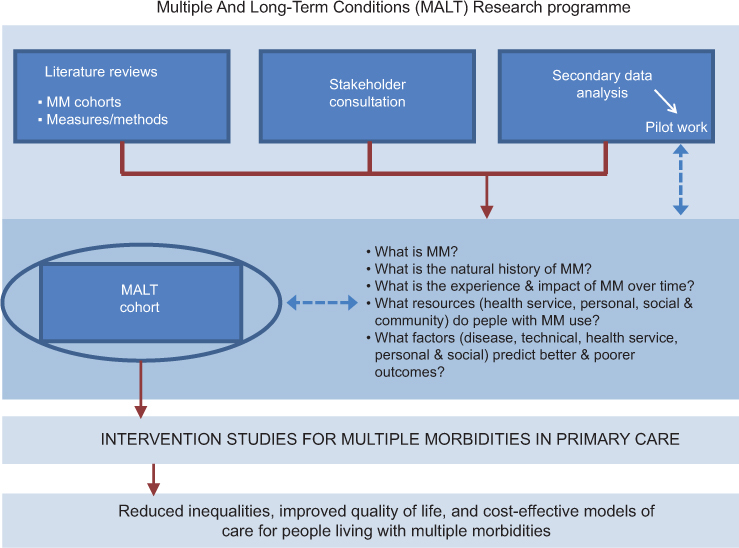
Research programme of the Scottish Multiple And Long-Term conditions (MALT) prospective, cohort study. MM, multimorbidity.

In developing the MALT study (see Figure 2), we have been assisted by a 2-year international Visiting Professor Award (J.G.) and funding for two part-time post-doctoral researchers by the Scottish School of Primary Care. Work to date has included a systematic review [27], ongoing secondary analysis of a nationally representative cross-sectional primary care database of 1.8 million people with up to 40 LTCs, and identification of existing longitudinal data amendable to secondary analysis. A series of stakeholder meetings have also been held with affiliated MALT researchers in Scotland, qualitative interviews with senior policy makers and NHS strategists, and a 1-day international workshop with experts in multimorbidity research from Canada, Australia, Ireland, England, and Scotland.

Key areas of discussion in these meetings included defining the primary aims of the prospective cohort study, how best to define and measure multimorbidity, and whether to include a wide range of conditions in order to be as inclusive as possible or to focus on a limited number of LTCs of key public health concern. Consideration was also given as to how best to capture the required data, including what can be measured from routinely collected data, data linkage issues, and what new data need to be collected by direct methods, including survey methods and longitudinal qualitative interviews. The importance of international collaboration was also emphasized so that studies carried out in one country might have relevance in other countries, with the possibility of linked cohorts to aid our global understanding of the common issues in multimorbidity and its management.

Central to all future work on multimorbidity must be a focus on the patient as a functioning person, integrated within a family and a community. Nowhere in medicine is person-centred care more appropriate than in individuals with multimorbidity, with their complex physical and psychosocial needs [9, 10, 13, 31], and yet there has been surprisingly little research on this in this group in terms of patients’ expectations, the doctor–patient relationship, the process and outcomes of clinical encounters, and the importance of human aspects of care such as practitioner empathy [32].

## The need for prospective cohort studies

In conclusion, multimorbidity now represents the norm not the exception in individuals with LTCs, and is arguably the biggest challenge facing healthcare systems globally. Strong primary care systems are required to deliver the holistic, person-centred care that the complexity of multimorbidity demands. However, the evidence-based for effective interventions in multimorbidity in primary care is weak, and prospective cohort studies are required to gain a better understanding of the natural history and trajectory of multimorbidity in different populations, and how healthcare and other services can best be configured to meet the needs of people with multimorbidity. Given the vertical paradigm that currently still dominates healthcare, medical education, and research, there is much work that needs to be done.
